# Spatial Proximity Between PD-L1(+) Tumor-Associated Macrophages and CD8(+) T Cells Influences Response to Atezolizumab Plus Bevacizumab in Hepatocellular Carcinoma

**DOI:** 10.3390/cancers18091422

**Published:** 2026-04-29

**Authors:** Takuto Nosaka, Masahiro Ohtani, Junki Yamashita, Yosuke Murata, Yu Akazawa, Tomoko Tanaka, Kazuto Takahashi, Tatsushi Naito, Yoshiaki Imamura, Kenji Koneri, Takanori Goi, Yasunari Nakamoto

**Affiliations:** 1 Second Department of Internal Medicine, Faculty of Medical Sciences, University of Fukui, Fukui 910-1193, Japan; mohtani@u-fukui.ac.jp (M.O.); yamaj@u-fukui.ac.jp (J.Y.); yosukem@u-fukui.ac.jp (Y.M.); aka0124@u-fukui.ac.jp (Y.A.); kawakami@u-fukui.ac.jp (T.T.); tkazuto@u-fukui.ac.jp (K.T.); naitot@u-fukui.ac.jp (T.N.); 2 Division of Diagnostic Pathology/Surgical Pathology, University of Fukui Hospital, Fukui 910-1193, Japan; suki@u-fukui.ac.jp; 3 First Department of Surgery, Faculty of Medical Sciences, University of Fukui, Fukui 910-1193, Japan; koneri@u-fukui.ac.jp (K.K.); tgoi@u-fukui.ac.jp (T.G.)

**Keywords:** hepatocellular carcinoma, spatial immune interactions, tumor-associated macrophages, CD8(+) T cells, atezolizumab plus bevacizumab

## Abstract

Background: Responses to atezolizumab plus bevacizumab (Atezo+Bev) in hepatocellular carcinoma (HCC) are heterogeneous, and response determinants remain unclear. We investigated whether spatial proximity between PD-L1(+) tumor-associated macrophages (TAMs) and CD8(+) T cells represents an immune niche associated with Atezo+Bev responsiveness. Methods: Multiplex immunohistochemistry was performed on biopsies from patients treated with Atezo+Bev (*n* = 23) or lenvatinib (*n* = 20). An interaction variable was defined via nearest-neighbor analysis as CD8(+) T cells within 25 µm of PD-L1(+) TAMs, normalized to cell counts. Associations with tumor shrinkage and progression-free survival (PFS) were examined. CD8(+) T cell phenotypes were evaluated via GZMB and TIM3. Transcriptomic profiling of resected HCCs (*n* = 8) was conducted using next-generation sequencing and gene set enrichment analysis (GSEA). Results: In a patient with responsive and non-responsive lesions, the responsive lesion showed closer PD-L1(+) TAM-CD8(+) T cell proximity. In cohort analyses, the interaction variable was associated with tumor shrinkage and prolonged PFS in the Atezo+Bev group, whereas PD-L1(+) TAM or CD8(+) T cell density alone was not predictive. This association was absent in the lenvatinib cohort. High-interaction tumors showed increased GZMB(+) and TIM3(+) CD8(+) T cells. Transcriptomic analysis revealed the upregulation of inflammatory, cytotoxic, chemotactic, and immunoregulatory genes, with enrichment of the chemokine, IFN-gamma, and IL-10 signaling pathways. Conclusions: Spatial proximity between PD-L1(+) TAMs and CD8(+) T cells defines an immune niche characterized by coexisting immune activation and regulatory programs and is strongly associated with Atezo+Bev responsiveness in HCC. Quantification of this spatial interaction may serve as a biopsy-based biomarker for immunotherapy stratification.

## Simple Summary

Responses to atezolizumab plus bevacizumab in hepatocellular carcinoma remain heterogeneous, and reliable predictive biomarkers are lacking. While most studies have focused on immune cell abundance, emerging evidence suggests that spatial organization within the tumor immune microenvironment plays a critical role. In this study, we demonstrate that the spatial proximity between PD-L1-positive tumor-associated macrophages and CD8-positive T cells is strongly associated with therapeutic response and progression-free survival, whereas the density of each cell type alone is not predictive. Tumors with high levels of this spatial interaction exhibit a transcriptional profile characterized by the coexistence of cytotoxic immune activation and immunoregulatory signaling, consistent with an adaptive immune resistance state. These findings highlight the importance of spatial immune architecture as a functional determinant of immunotherapy response and suggest that quantifying immune cell interactions in tumor biopsies may provide a clinically actionable biomarker for treatment stratification.

## 1. Introduction

Hepatocellular carcinoma (HCC) remains a malignancy with a poor prognosis, and the optimization of therapeutic strategies for advanced disease is still required [1,2]. In recent years, combination therapy with the anti-PD-L1 antibody atezolizumab and the anti-VEGF antibody bevacizumab (Atezo+Bev) has been established as a first-line treatment for advanced HCC, leading to significant improvements in progression-free and overall survival [3,4]. However, clinical responses to Atezo+Bev are highly heterogeneous, with a substantial proportion of patients exhibiting primary resistance or early disease progression [5]. Underlying liver disease etiology has been reported to influence the tumor immune microenvironment in HCC [1]. Chronic viral hepatitis (HBV/HCV) is associated with persistent antigen exposure and T cell exhaustion, whereas steatohepatitis-related HCC is characterized by metabolic inflammation and distinct immune dysregulation [6,7]. Alcohol-related liver disease may also exhibit unique immune alterations associated with chronic inflammation and immune dysfunction, potentially contributing to heterogeneity in tumor immune contexture [8]. The immunological determinants of this heterogeneity within the tumor immune microenvironment (TIME) remain incompletely understood.

The efficacy of immune checkpoint inhibitor (ICI) therapy is strongly influenced by the composition of the TIME and the functional states of immune cells [5,9]. Among these, tumor-associated macrophages (TAMs) represent a dominant immune population that contributes to HCC progression and the establishment of an immunosuppressive microenvironment [10,11,12]. In particular, PD-L1-expressing TAMs (PD-L1(+) TAMs) have emerged as key immunoregulatory cells capable of suppressing CD8(+) T cell-mediated antitumor immunity [13,14]. We previously demonstrated, using multiplex immunohistochemistry (mIHC), that PD-L1(+) TAMs induce CD8(+) T cell exhaustion and contribute to immunosuppression in HCC [15].

Recent advances in tumor immunology have highlighted that therapeutic efficacy is determined not only by immune cell abundance or phenotype, but also by their spatial organization and intercellular interactions within tumors [9,16]. Consistent with this concept, studies in melanoma and colorectal cancer have shown that spatial proximity between PD-L1-expressing myeloid cells and CD8(+) T cells is associated with responsiveness to ICI therapy [17,18,19]. Nevertheless, the relevance of such spatial immune interactions in the context of Atezo+Bev-treated HCC remains largely unexplored.

This study focused on the spatial interactions between PD-L1(+) TAMs and CD8(+) T cells as a potential feature of the TIME that may explain heterogeneity in responses to Atezo+Bev in HCC. Spatial proximity indices were quantified by integrating the mIHC of pretreatment tumor biopsy specimens with gene expression analyses of resected HCC samples, including targeted next-generation sequencing (NGS), and their associations with treatment response were examined. Consequently, tumors with high PD-L1(+) TAM–CD8(+) T cell interactions exhibited an immune niche characterized by the coexistence of immune activation and regulatory programs and were associated with favorable progression-free survival (PFS) and overall survival (OS) under Atezo+Bev therapy, supporting the clinical relevance of spatial immune architecture in HCC.

## 2. Materials and Methods

### 2.1. Study Protocol and Patients

A retrospective cohort was assembled comprising patients with HCC who underwent systemic treatment with Atezo+Bev or lenvatinib at the University of Fukui Hospital between May 2018 and February 2025. During this period, 131 patients were identified as potential candidates (Atezo+Bev, *n* = 55; lenvatinib, *n* = 76). Eligibility for the present analysis required the availability of pretreatment percutaneous liver tumor biopsy specimens (*n* = 46). Patients were excluded if clinical information or assessment of therapeutic response was insufficient (*n* = 3). Following application of these criteria, a total of 43 patients were included in the final study population (Atezo+Bev, *n* = 23; lenvatinib, *n* = 20). Liver tumor biopsy samples and serum specimens were obtained prior to the initiation of systemic therapy. Baseline demographic data and clinical characteristics of the enrolled patients are provided in Table 1. The diagnosis of HCC was established using contrast-enhanced imaging in accordance with the clinical practice guidelines of the American Association for the Study of Liver Diseases, and/or histopathological confirmation from a liver tumor biopsy. This study was conducted in accordance with the Declaration of Helsinki and was approved by the Research Ethics Committee of the Faculty of Medicine, University of Fukui (Approval No.: 20200086; Approval Date: 20 August 2020). Written informed consent was obtained from all patients prior to participation.

### 2.2. Etiology of Liver Diseases

The etiology of HCC was determined based on viral hepatitis markers. Cases positive for anti-hepatitis C virus antibodies (anti-HCV Ab) were classified as hepatitis C virus (HCV)-related HCC, whereas cases positive for hepatitis B surface antigen (HBsAg) were classified as hepatitis B virus (HBV)-related HCC. Patients who were negative for both anti-HCV Ab and HBsAg were categorized as non-B, non-C (NBNC) HCC.

### 2.3. Treatment Regimens of Atezo+Bev and Lenvatinib

Atezolizumab (1200 mg) in combination with bevacizumab (15 mg/kg) was administered at three-week intervals in accordance with the IMbrave150 study regimen [4]. In the event of treatment-related adverse events (AEs), interruption or dose modification of atezolizumab and/or bevacizumab was undertaken as clinically indicated. Lenvatinib was prescribed orally at a daily dose of 8 mg for patients with a body weight of less than 60 kg and 12 mg for those weighing 60 kg or more [20]. Dose reduction or temporary suspension of lenvatinib was implemented when grade 2 AEs were considered intolerable or when grade 3 AEs occurred. The choice of systemic therapy was made in accordance with established clinical guidelines and multidisciplinary discussions at the institution, and the final treatment strategy was determined through shared decision-making between the attending physician and the patient. Patient management was conducted by a multidisciplinary team, including hepatologists, oncologists, radiologists, and, when appropriate, nutritional support specialists. Treatment was discontinued upon the occurrence of unacceptable toxicity or radiologically confirmed progressive disease (PD). All adverse events were assessed and graded using the National Cancer Institute Common Terminology Criteria for Adverse Events (CTCAE), version 5.0.

### 2.4. Evaluation of Treatment Efficacy

Assessment of the initial therapeutic response was performed between 8 and 12 weeks after treatment initiation using either dynamic computed tomography (CT) or gadoxetic acid-enhanced magnetic resonance imaging (Gd-EOB–MRI). Thereafter, follow-up imaging studies were conducted at intervals of 6 to 12 weeks. Radiological response was determined in accordance with the Response Evaluation Criteria in Solid Tumors (RECIST), version 1.1. Progression-free survival (PFS) was defined as the time from the start of systemic therapy to the date of radiologically confirmed disease progression or death from any cause. Overall survival (OS) was defined as the duration from treatment initiation to death from any cause.

### 2.5. Assessment of Hepatic Reserve Function

The albumin–bilirubin (ALBI) score was derived using the following equation: ALBI score = [log10 total bilirubin (µmol/L) × 0.66] + [albumin (g/L) × −0.085]. Patients were categorized according to the calculated ALBI score into three grades: Grade 1 (≤−2.60), Grade 2 (>−2.60 and ≤−1.39), and Grade 3 (>−1.39) [21]. Grade 2 was further stratified into two subgroups, Grade 2a and Grade 2b, based on a previously reported cutoff value of −2.270. These four classifications were collectively defined as the modified ALBI (mALBI) grades [22]. In addition, the Child–Pugh classification was also evaluated as a conventional indicator of hepatic functional reserve [23].

### 2.6. Percutaneous Hepatic Tumor Needle Biopsy

Tumor tissue samples were collected from patients with HCC prior to the initiation of Atezo+Bev or lenvatinib therapy. Percutaneous needle biopsy targeting the intratumoral core was performed under abdominal ultrasonographic guidance using a 21-gauge Majima needle (TOP Corporation, Tokyo, Japan). The obtained specimens were subsequently fixed in formalin and processed for paraffin embedding. Tumor tissue was obtained using 3–4 percutaneous needle biopsy cores per patient, targeting the intratumoral region under ultrasonographic guidance. Areas with extensive necrosis or dense fibrosis were excluded, and only regions with preserved cellular architecture were included in the spatial analysis. In selected cases, including the representative case presented in the Results section, spatial immune analyses were performed using biopsy specimens obtained at different time points to illustrate intrapatient heterogeneity.

### 2.7. Multiplexed Immunofluorescence Staining

Multiplex immunohistochemistry was performed using the Opal Multiplex IHC platform (PerkinElmer/Akoya Biosciences, CA, USA) with minor modifications from previously reported protocols [15]. Briefly, formalin-fixed paraffin-embedded tumor sections (4 µm) were subjected to antigen retrieval using AR6 or AR9 buffer, followed by sequential staining with antibodies against CD8/Opal 520, TIM3/Opal 540, Granzyme B/Opal 570, PD-L1/Opal 620, CD163/Opal 650, and CD68/Opal 690 using tyramide signal amplification. Whole-slide images were acquired with the Mantra^®^ system. Multiplex immunofluorescence images were analyzed using inForm^®^ software v2.6 (Akoya Biosciences, CA, USA). Cell segmentation was performed based on DAPI nuclear staining, where nuclear morphology and size were used to identify individual cells, followed by expansion to define cytoplasmic and membranous compartments. Marker positivity for PD-L1, CD68, CD163, and CD8 was determined using a supervised machine learning-based classification algorithm within inForm^®^. The classifier was trained using representative positive and negative cells selected from multiple regions of interest. This approach incorporates mean fluorescence intensity (MFI) and subcellular localization patterns, functioning as a multivariate classification system rather than relying on a single fixed cutoff. Cells exhibiting fluorescence signals above the background levels were classified as positive for CD8, CD68, and CD163, while PD-L1 positivity was defined based on membranous and cytoplasmic expression patterns. To ensure consistency, fluorescence intensity distributions across slides were reviewed, and the classification model was calibrated. Cell phenotyping was performed using the phenoptr^®^ package, and classification results were visually validated to confirm concordance with histopathological features. Detailed staining conditions and reagents are provided in Supplementary Table S1.

### 2.8. Interaction Variable

Intercellular interaction variables between immune cell populations were computed based on previously published approaches [15,24]. Spatial analyses, including nearest-neighbor distance measurements and the computation of interaction variables, were conducted using the phenoptr^®^ package. To capture direct cell–cell contact and short-range paracrine interactions, a 25 µm radius was selected, corresponding approximately to one to two cell diameters. This distance is consistent with prior spatial immunology studies using similar proximity thresholds [25,26]. For each image, we counted the number of CD8(+) T cells that were located within a 25 µm radius of at least one PD-L1(+) TAM, and this value was defined as the PD-L1(+) TAM–CD8(+) T cell interaction variable. To minimize the influence of the absolute abundance of either CD8(+) T cells or PD-L1(+) TAMs alone, this value was normalized by dividing it by the sum of CD8(+) T cells and PD-L1(+) TAMs present within the corresponding image region and then multiplying by 100 (a schematic illustration of the interaction variable is shown in Supplementary Figure S1). For Kaplan–Meier survival analyses, samples were dichotomized into high- and low-interaction groups using the median value of the interaction variable as the cutoff.

### 2.9. Serum Cytokine Measurement

Immediately before the initial administration of atezolizumab plus bevacizumab or lenvatinib, peripheral venous blood samples were collected, and serum was separated via centrifugation at 1000× *g* for 5 min. The isolated serum samples were stored at −80 °C until analysis. Serum cytokine concentrations were measured using the BD™ Cytometric Bead Array (CBA) Human Inflammatory Cytokine Kit I (BD Biosciences, Franklin Lakes, NJ, USA) in accordance with the manufacturer’s instructions and previously reported methods. Briefly, capture beads coated with cytokine-specific antibodies were incubated with serum samples and detection reagents at room temperature for 3 h. After washing, cytokine–bead complexes were analyzed using a FACSymphonyTM A1 flow cytometer (BD Biosciences), and data analysis was performed with BD CBA Analysis Software v1.1.15 (BD Biosciences).

### 2.10. Analysis of Human Tissue Samples

Between April 2006 and January 2024, eight patients with HCC who underwent hepatectomy at the University of Fukui Hospital were included in this analysis (Table 2). Histological evaluation was performed using the resected specimens. This study was conducted in accordance with the Declaration of Helsinki and was approved by the Research Ethics Committee of the University of Fukui (Approval No.: 20210168; Approval Date: 18 January 2022).

### 2.11. RNA Extraction and Next-Generation Sequencing from FFPE Specimens of Resected Hepatocellular Carcinoma

Formalin-fixed paraffin-embedded (FFPE) specimens were sectioned at a thickness of 4 µm. Tumor regions were identified and dissected with reference to hematoxylin and eosin (H&E)-stained slides. Total RNA was extracted using the RNeasy FFPE Kit (QIAGEN, Hilden, Germany). RNA quantity and quality were assessed using a Qubit 3.0 Fluorometer with the RNA HS Assay (Thermo Fisher Scientific, Waltham, MA, USA), and RNA integrity was evaluated using the Bioanalyzer 2100 (Agilent Technologies, Santa Clara, CA, USA). RNA sequencing was performed using custom AmpliSeq for Illumina RNA panels (Illumina, San Diego, CA, USA) consisting of two gene panels targeting 373 cancer progression-related genes and 240 cancer immunity-related genes, thereby restricting the analysis to a pre-selected set of genes. Library preparation was initiated with 100 ng of input RNA. Library construction and complementary DNA (cDNA) synthesis were performed according to the manufacturer’s instructions. cDNA synthesis was carried out using the AmpliSeq cDNA Synthesis for Illumina (Illumina, San Diego, CA, USA). RNA libraries were prepared using custom AmpliSeq for Illumina RNA panels consisting of two gene panels targeting 373 cancer progression-related genes and 240 cancer immunity-related genes. Library preparation was performed using the AmpliSeq Library PLUS for Illumina and AmpliSeq CD Indices Set A for Illumina (Illumina). RNA sequencing was performed on a MiSeq system (Illumina) using the MiSeq Reagent Kit v3 to generate 2 × 150 bp paired-end reads. FASTQ files generated by sequencing were automatically analyzed using the Local Run Manager RNA Amplicon Analysis Module (Illumina). The raw sequencing data have been deposited in the NCBI Gene Expression Omnibus (GEO) database under accession number GSE318324.

### 2.12. Gene Expression and Pathway Analysis

Statistical analyses were performed in R (version 4.4) using RNA-Seq read count data in CSV format. Duplicate gene symbols were aggregated by summing their corresponding read counts. Lowly expressed genes were filtered by retaining genes with a total read count of at least one across all eight samples. Data normalization was conducted using the trimmed mean of M values (TMM) method implemented in the edgeR package, which adjusts for library size and compositional differences even in targeted RNA sequencing data. Normalized expression levels were represented as log_2_-transformed counts per million (logCPM). Differential gene expression (DGE) analysis was performed between two groups stratified according to an interaction variable (High group, *n* = 4; Low group, *n* = 4). Following dispersion estimation with edgeR, between-group differences in gene expression were assessed using an exact test. Statistical significance was defined as a *p* value < 0.05 and an absolute log_2_ fold change > 1.0. For data visualization, heatmaps based on row-scaled logCPM values of all significantly differentially expressed genes (*p* < 0.05) were generated using hierarchical clustering, together with volcano plots illustrating global expression changes. For functional analysis, gene set enrichment analysis (GSEA) was performed using the Reactome database. All genes were ranked according to their log fold change values, and enrichment analysis was conducted using the clusterProfiler v4.16.0 package. Pathway significance was evaluated based on normalized enrichment scores (NES) and *p* values.

### 2.13. Statistical Analyses

Differences between groups were assessed using the Mann–Whitney U test, Fisher’s exact test, chi-square test, or one-way analysis of variance (ANOVA) followed by the Tukey–Kramer multiple comparison procedure, as appropriate. Survival outcomes were evaluated using the Kaplan–Meier method, and statistical differences between groups were examined with the log-rank test. To identify clinicopathological variables associated with survival, Cox proportional hazards regression models were applied. Variables showing a *p* value of less than 0.05 in univariate analyses were subsequently entered into multivariate models. The predictive performance of biomarkers was evaluated by receiver-operating characteristic (ROC) curve analysis, and the optimal cutoff values were determined using Youden’s index (sensitivity + specificity − 1).

Internal validation was performed using bootstrap resampling (1000 iterations). For the Cox proportional hazards model, the optimism-corrected Harrell’s concordance index (C-index) was estimated using the rms package in R. For ROC analysis, a logistic regression model was constructed for 1-year event prediction, and the optimism-corrected area under the curve (AUC) was estimated using bootstrap resampling with the pROC package.

All statistical analyses were performed using GraphPad Prism version 10.5.0 (GraphPad Software Inc., San Diego, CA, USA) and R software (version 4.4.2; R Foundation for Statistical Computing, Vienna, Austria). A two-sided *p* value < 0.05 was considered indicative of statistical significance. Comparisons not explicitly shown in the figures were regarded as not statistically significant.

## 3. Results

### 3.1. Intrapatient Spatial Immune Differences in Atezo+Bev-Treated HCC

**Figure 1:**
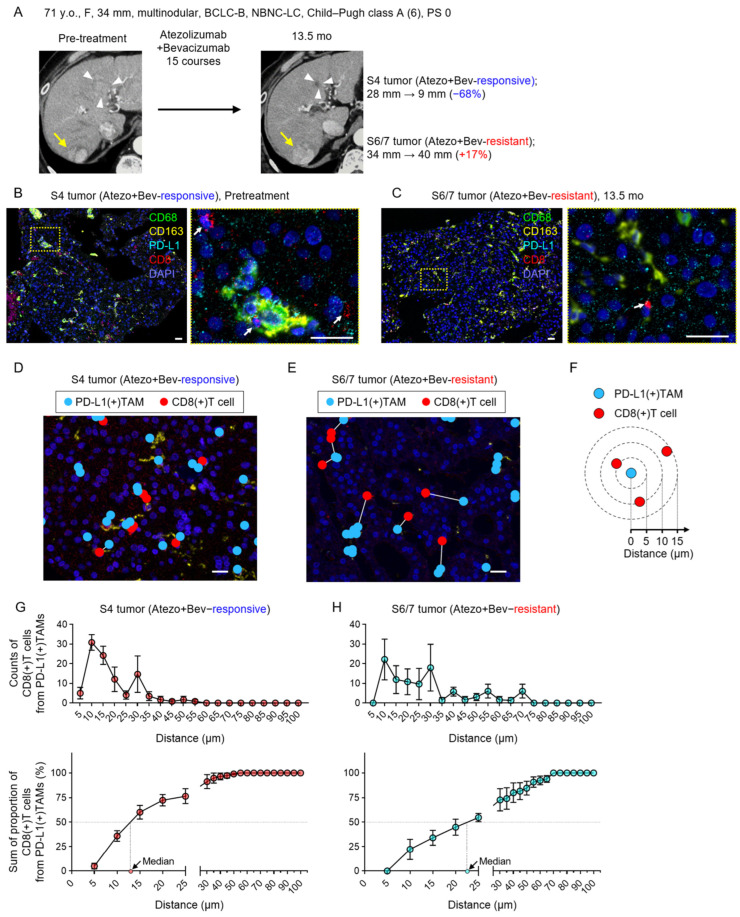
A representative HCC case showing distinct spatial PD-L1(+) TAM–CD8 T cell interactions in Atezo+Bev-responsive and -resistant lesions. (**A**) A representative clinical course of a patient with hepatocellular carcinoma (HCC) treated with atezolizumab plus bevacizumab (Atezo+Bev). Contrast-enhanced CT images in the arterial phase obtained before treatment (left) and 13.5 months after initiation of Atezo+Bev (right) are shown. The S4 tumor exhibited a response to Atezo+Bev, whereas the S6/7 tumor was resistant to Atezo+Bev. White arrowheads indicate the S4 tumor, and yellow arrows indicate the S6/7 tumor. (**B**,**C**) Multiplex immunofluorescence staining of immune cell markers in HCC biopsy specimens (DAPI, blue; CD68, green; CD163, yellow; PD-L1, cyan; CD8, red). White arrows indicate CD8(+) T cells. Scale bar, 25 µm. A pretreatment specimen from the Atezo+Bev-responsive S4 tumor (**B**) and a specimen obtained 13.5 months after treatment initiation from the Atezo+Bev-resistant S6/7 tumor (**C**) are shown. The dashed yellow box indicates the region magnified in the adjacent panel. (**D**,**E**) Nearest-neighbor mapping of PD-L1(+) tumor-associated macrophages (TAMs; blue) and CD8(+) T cells (red) in tumor tissues. Scale bar, 25 µm. The Atezo+Bev-responsive S4 tumor (**D**) and the Atezo+Bev-resistant S6/7 tumor (**E**) are shown. (**F**) A schematic diagram illustrating the number and spatial distribution of CD8(+) T cells measured at 5 µm intervals from PD-L1(+) TAMs. (**G**,**H**) Numbers (upper panels) and summed proportions (lower panels) of CD8(+) T cells according to the distance from PD-L1(+) TAMs. Data are presented as mean ± standard deviation. The Atezo+Bev-responsive S4 tumor (**G**) and the Atezo+Bev-resistant S6/7 tumor (**H**) are shown.

A 71-year-old woman was found to have HCC lesions measuring up to 28 mm in segment 4 (S4) and 34 mm in segments 6/7 (S6/7). After 15 cycles of Atezo+Bev therapy, the S4 lesion showed tumor shrinkage (68%), whereas the S6/7 lesion increased in size (17%) (Figure 1A). Multiplex IHC analysis demonstrated a clear difference in spatial organization between the two lesions (Figure 1B–E). The median distance from PD-L1(+) TAMs to the nearest CD8(+) T cells was 13 µm in the responsive S4 lesion and 23 µm in the resistant S6/7 lesion (Figure 1F–H). In this case, both responsive and resistant lesions to Atezo+Bev therapy were observed simultaneously, and the responsive tumor exhibited a higher degree of spatial proximity between PD-L1(+) TAMs and CD8(+) T cells. The biopsy specimen from the resistant S6/7 lesion was obtained 13.5 months after treatment initiation; therefore, treatment-induced changes in the tumor microenvironment, including stromal remodeling, necrosis, or altered immune cell infiltration, may have influenced the observed spatial patterns. This case is presented for illustrative purposes, and all cohort-based analyses were conducted using pretreatment biopsy specimens.

### 3.2. Pretreatment Factors Associated with Therapeutic Response to Atezo+Bev and Lenvatinib

**Figure 2:**
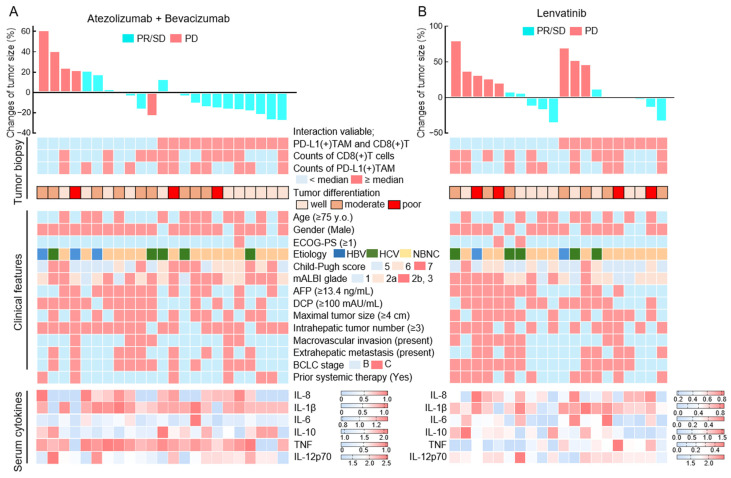
Pretreatment immune, clinical, and cytokine profiles associated with tumor response in Atezo+Bev- and lenvatinib-treated HCC. (**A**,**B**) Heatmaps showing tumor size changes (%) and pretreatment factors in 23 patients with hepatocellular carcinoma (HCC) treated with atezolizumab plus bevacizumab (**A**) and in 20 patients with HCC treated with lenvatinib (**B**). Pretreatment factors include (1) immunohistochemical evaluation of PD-L1(+) tumor-associated macrophages (TAMs) and CD8(+) T cells in liver tumor biopsy specimens, (2) clinical characteristics, and (3) serum cytokine concentrations.

Among the 23 patients with HCC treated with Atezo+Bev, 18 patients achieved a partial response (PR) or stable disease (SD), while 5 patients showed progressive disease (PD) at the first radiological assessment (Figure 2A). In contrast, among the 20 patients treated with lenvatinib, 12 patients achieved PR or SD, whereas 8 patients exhibited PD at the initial response evaluation (Figure 2B). The following pretreatment factors were analyzed for their association with initial therapeutic response to Atezo+Bev or lenvatinib: (1) interactions, cell density, and differentiation status of PD-L1(+) TAMs and CD8(+) T cells in tumor biopsy specimens; (2) clinical background characteristics; and (3) inflammation-related serum cytokines (Supplementary Figure S1). In the univariate analysis, the interaction between PD-L1(+) TAMs and CD8(+) T cells within tumor tissue, DCP levels, and vascular invasion was significantly associated with PFS in patients treated with Atezo+Bev. The multivariate analysis demonstrated that the interaction between PD-L1(+) TAMs and CD8(+) T cells, as well as vascular invasion, was independently associated with PFS. No significant associations were observed for any of the other evaluated factors in either the Atezo+Bev-treated or lenvatinib-treated cohorts (Table 3 and Table S2).

### 3.3. Spatial Interactions Between PD-L1(+) TAMs and CD8(+) T Cells and Therapeutic Response

**Figure 3:**
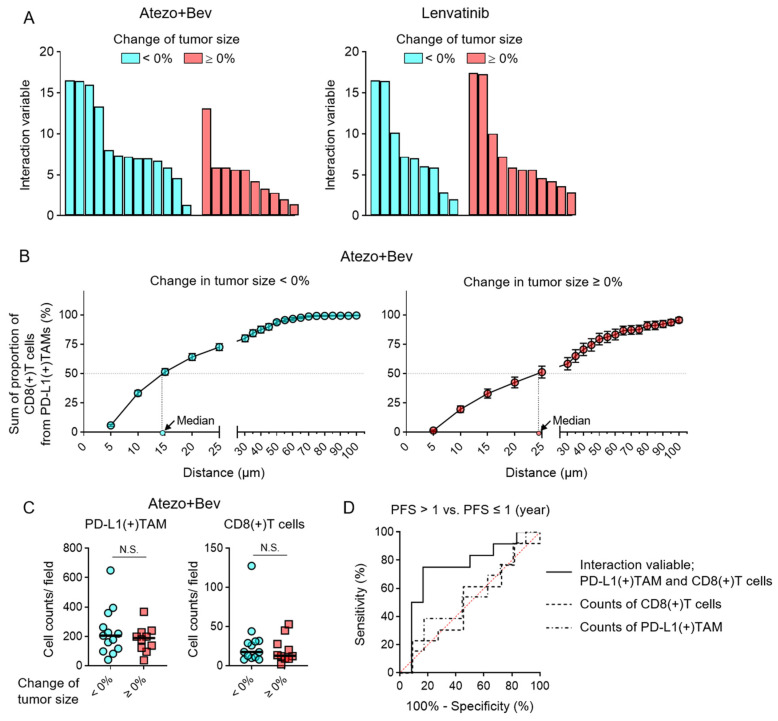
Pretreatment PD-L1(+) TAM–CD8(+) T cell interactions and tumor response to systemic therapy in HCC. (**A**) Tumor size changes at the time of the first radiological response assessment and the interaction variable between PD-L1(+) tumor-associated macrophages (TAMs) and CD8(+) T cells in tumor biopsy specimens from patients treated with atezolizumab plus bevacizumab (Atezo+Bev) or lenvatinib. (**B**) Association between tumor size changes at the first radiological response assessment and the sum of the proportion of CD8(+) T cells in proximity to PD-L1(+) TAMs (%) in Atezo+Bev-treated patients. (**C**) Association between tumor size changes at the first radiological response assessment and the absolute numbers of PD-L1(+) TAMs and CD8(+) T cells in tumor biopsy specimens from Atezo+Bev-treated patients. (**D**) Predictive performance of the PD-L1(+) TAM–CD8(+) T cell interaction variable for progression-free survival (PFS), compared with the absolute numbers of each cell population. Receiver operating characteristic (ROC) curves are shown for discrimination between patients with PFS > 1 year and PFS ≤ 1 year. (**A**,**C**) Mann–Whitney U test. N.S., not significant.

The association between the interaction variable of PD-L1(+) TAMs and CD8(+) T cells within tumor tissue and changes in tumor size at the first radiological assessment following systemic therapy was evaluated. In patients treated with Atezo+Bev, cases exhibiting tumor shrinkage showed significantly higher interaction variable values than those with tumor progression (Figure 3A). In contrast, no significant association was observed between tumor size changes and the interaction variable in patients treated with lenvatinib. The median distance from PD-L1(+) TAMs to the nearest CD8(+) T cells was 14 µm in patients with tumor shrinkage, compared with 24 µm in those with tumor progression (Figure 3B). Nearest-neighbor distance analyses likewise demonstrated significantly shorter distances between PD-L1(+) TAMs and CD8(+) T cells in responsive tumors. In contrast, neither the number of PD-L1(+) TAMs nor the number of CD8(+) T cells was associated with therapeutic response (Figure 3C). Furthermore, receiver operating characteristic (ROC) analysis discriminating PFS >1 year from PFS ≤1 year demonstrated that the PD-L1(+) TAM–CD8(+) T cell interaction variable yielded a significantly higher area under the ROC curve (AUROC), whereas PD-L24 April 2026+) TAM counts and CD8(+) T cell counts showed no significant discriminatory ability (Figure 3D). Internal validation demonstrated an apparent AUC of 0.881 and an optimism-corrected AUC of 0.768, indicating that the model retained reasonable discriminative performance after correction for optimism. To ensure that the prognostic value of the interaction variable was not dependent on the specific distance threshold, we performed a sensitivity analysis using alternative spatial radii (15, 30, and 50 µm). As shown in Supplementary Figure S2, the interaction variables calculated at these different distances were highly correlated (Spearman’s ρ range: 0.87–0.99, all *p* < 0.001), demonstrating strong consistency across spatial scales. Furthermore, the direction and statistical significance of the associations with clinical outcomes were preserved across all tested radii. Notably, hazard ratios for the interaction variable remained consistently below 1.0, with all 95% confidence intervals not crossing the null value, and significant associations with progression-free survival were observed at each distance threshold (Supplementary Figure S3). Collectively, these results indicate that spatial interactions between PD-L1(+) TAMs and CD8(+) T cells within the tumor microenvironment may reflect therapeutic responsiveness and prognosis in patients treated with Atezo+Bev.

### 3.4. Spatial Interactions Between PD-L1(+) TAMs and CD8(+) T Cells and Functional Marker Expression

**Figure 4:**
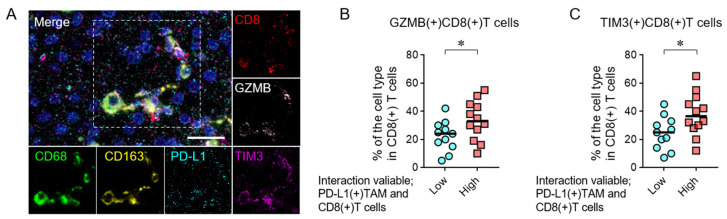
Functional states of CD8(+) T cells stratified by PD-L1(+) TAM–CD8(+) T cell spatial interactions. (**A**) Representative multiplex immunofluorescence images of immune cell markers in HCC biopsy specimens. Nuclei are stained with DAPI (blue) in the merged image, CD68 (green), CD163 (yellow), PD-L1 (cyan), CD8 (red), granzyme B (GZMB; white), and TIM3 (purple). The dashed box indicates the region used for signal analysis of each marker. Scale bar, 25 µm. (**B**,**C**) Comparison of the proportions of intratumoral CD8(+) T cells expressing GZMB (**B**) and TIM3 (**C**). Patients were stratified into high (Int.val. High, *n* = 12) and low (Int.val. Low, *n* = 11) groups based on the interaction variable between PD-L1(+) TAMs and CD8(+) T cells in tumor regions. (**B**,**C**) Mann–Whitney U test. * *p* < 0.05.

To evaluate the association between the interaction variable of PD-L1(+) TAMs and CD8(+) T cells and the functional phenotypes of CD8(+) T cells, mIHC was performed to assess the expression of Granzyme B (GZMB) and TIM3 as functional markers (Figure 4A). Cases with a high interaction variable exhibited a significantly higher proportion of GZMB-expressing CD8(+) T cells (Figure 4B). In addition, the proportion of TIM3-expressing CD8(+) T cells was also significantly higher in cases with a high interaction variable (Figure 4C). These findings suggest that spatial interactions between PD-L1(+) TAMs and CD8(+) T cells may be associated with both the activation- and exhaustion-related phenotypes of CD8(+) T cells.

### 3.5. Association Between PD-L1(+) TAM–CD8(+) T Cell Interactions and Clinical Outcomes

**Figure 5:**
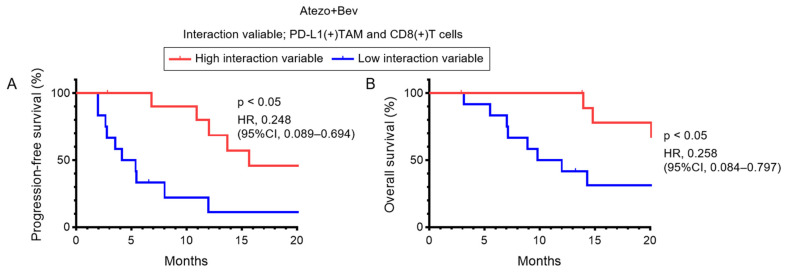
PD-L1(+) TAM–CD8(+) T cell interaction stratifies survival outcomes in Atezo+Bev-treated HCC. (**A**,**B**) Kaplan–Meier curves for progression-free survival (PFS) (**A**) and overall survival (OS) (**B**) in patients treated with atezolizumab plus bevacizumab (Atezo+Bev). Patients were stratified into high (Int.val. High, *n* = 12) and low (Int.val. Low, *n* = 11) groups based on the interaction variable between PD-L1(+) tumor-associated macrophages (TAMs) and CD8(+) T cells. (**A**,**B**) Log-rank test. CI, confidence interval; HR, hazard ratio.

In patients treated with Atezo+Bev, the association between the interaction variable of intratumoral PD-L1(+) TAMs and CD8(+) T cells and clinical outcomes was analyzed. Patients with a high interaction variable exhibited significantly longer PFS than those with a low interaction variable (*p* < 0.05; hazard ratio [HR] = 0.248; 95% confidence interval [CI], 0.089–0.694) (Figure 5A). OS was also significantly improved in patients with a high interaction variable (*p* < 0.05; HR = 0.258; 95% CI, 0.084–0.797) (Figure 5B). These findings suggest that the intratumoral interaction variable between PD-L1(+) TAMs and CD8(+) T cells may serve as a prognostic indicator of therapeutic outcomes in patients receiving Atezo+Bev.

### 3.6. Intratumoral Molecular Profiles Associated with PD-L1(+) TAM–CD8(+) T Cell Interactions in HCC

**Figure 6:**
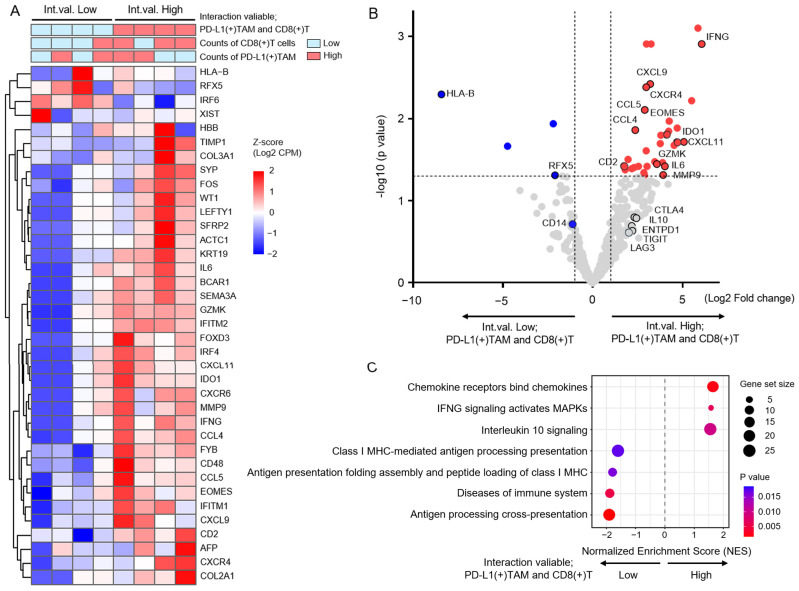
Molecular expression profiles and immune pathway analyses associated with PD-L1(+) TAM–CD8(+) T cell interactions. (**A**) Heatmap integrating the histological evaluation of PD-L1(+) tumor-associated macrophages (TAMs), CD8(+) T cells, and RNA expression profiles obtained via NGS in tumor tissues from eight resected liver specimens. Cases were stratified into high (*n* = 4) and low (*n* = 4) groups according to the PD-L1(+) TAM–CD8(+) T cell interaction variable, and genes showing statistically significant differential expression between the two groups are displayed. Gene expression levels are presented as Z-scores (log2 CPM). (**B**) Volcano plot comparing gene expression between the two groups stratified by the PD-L1(+) TAM–CD8(+) T cell interaction variable. Gene names of immune function-related molecules with statistically significant differences are indicated. Red circles indicate significantly upregulated genes, blue circles indicate significantly downregulated genes, and grey dots represent genes without significant changes. (**C**) Gene set enrichment analysis (GSEA) comparing the two groups defined by the PD-L1(+) TAM–CD8(+) T cell interaction variable. Enrichment results for immune-related Reactome pathways are shown.

To elucidate the intratumoral molecular profiles associated with the interaction between PD-L1(+) TAMs and CD8(+) T cells, we analyzed the patients with HCC who underwent surgical resection. RNA was extracted from tumor regions of sectioned FFPE samples, and the expression of 613 genes related to cancer progression was comprehensively analyzed by NGS (Figure 6A). In tumors with a high interaction variable, the expression of cytotoxic and inflammatory genes such as *IFNG* and *GZMK*, as well as chemokines including *CXCL9*, *CXCL11*, *CCL4*, and *CCL5*, was significantly increased, together with the upregulation of immune regulatory genes such as *IL6*, *EOMES*, *IDO1*, and *CD2*. T cell exhaustion markers, including *CTLA4*, *TIGIT*, and *LAG3*, showed a trend toward higher expression (Figure 6B). In contrast, higher expression of *HLA-B* and the transcriptional regulator *RFX5* was observed in tumors with a low interaction variable. GSEA revealed that tumors with a high interaction variable were enriched in immune response- and inflammation-related pathways, including chemokine receptors that bind chemokines, IFNG signaling that activates MAPKs, and interleukin 10 signaling (Figure 6C). In contrast, tumors with a low interaction variable showed significant enrichment of antigen presentation-related pathways, including antigen processing, cross-presentation, and antigen presentation folding assembly and peptide loading of class I MHC. These results suggest that the spatial interaction between PD-L1(+) TAMs and CD8(+) T cells may contribute to the formation of a tumor immune microenvironment characterized by the coexistence of inflammatory chemokine- and IFN-γ-dependent immune responses and immunosuppressive signaling.

## 4. Discussion

This study demonstrates that the spatial interaction between PD-L1(+) TAMs and CD8(+) T cells is strongly associated with therapeutic response and PFS in HCC patients treated with Atezo+Bev, whereas this association was not observed with lenvatinib. Tumors with high interaction levels were characterized by a microenvironment in which cytotoxic immune activation and immune exhaustion coexisted. Transcriptomic analysis further showed that these interactions were associated with coordinated activation of inflammatory and IFN-γ-related pathways together with the upregulation of immunosuppressive programs, suggesting that this spatial immune context may contribute to the clinical efficacy of Atezo+Bev.

The spatial interaction between PD-L1(+) TAMs and CD8(+) T cells consistently predicted initial treatment response in patients receiving Atezo+Bev. This finding supports the concept that effective antitumor immunity depends not only on immune cell abundance, but also on their spatial organization within the tumor microenvironment [16]. Regarding the clinical background, we observed no significant difference in the interaction variable based on the underlying liver disease etiology (HBV, HCV, or non-viral; *p* = 0.231 in Table 3). While chronic inflammation and immune landscapes differ among etiologies, our findings suggest that the spatial organization of PD-L1(+) TAMs and CD8(+) T cells may represent a common immunopathological feature associated with responsiveness to immune checkpoint inhibitors. Because PD-L1 expression can be induced by IFN-γ during active immune responses, close proximity between PD-L1(+) TAMs and CD8(+) T cells may reflect an immunologically active tumor niche associated with responsiveness to immune checkpoint inhibitor therapy, as reported in melanoma and colorectal cancer [17,18,19,27]. In our transcriptomic analysis, the high-interaction group exhibited concomitant upregulation of inflammatory and cytotoxic genes (e.g., IFNG and GZMK) together with immunoregulatory genes (e.g., IDO1 and IL6). In addition, enrichment of IFN-γ- and IL-10-related signaling pathways by GSEA supports the interpretation that this spatially proximal niche represents a site of adaptive immune resistance in which effector activity and suppressive feedback coexist.

The establishment of a microenvironment characterized by close proximity between PD-L1(+) TAMs and CD8(+) T cells may involve chemokine-mediated local recruitment [28,29]. We previously reported that PD-L1(+) TAMs in HCC highly express the CXCR3 ligands CXCL9, CXCL10, and CXCL11, and that CXCR3(+) CD8(+) T cells preferentially accumulate near these TAMs [15]. Our NGS analysis consistently revealed significant upregulation of CXCL9, CXCL11, CCL4, and CCL5, along with enrichment of the “chemokine receptor binding” pathway. Together, these results suggest that PD-L1(+) TAMs may promote functional spatial immune clusters by recruiting and retaining CXCR3(+) CD8(+) T cells through chemokine production, consistent with an IFN-γ-dependent inflammatory niche associated with adaptive immune resistance [27,30]. In contrast, even in tumors with limited spatial proximity between PD-L1(+) TAMs and CD8(+) T cells, CD8(+) T cell numbers were preserved and were not associated with therapeutic efficacy, indicating that intratumoral presence alone does not necessarily translate into effective functional interactions. The interaction variable was normalized to reflect the relative frequency of interacting cells rather than absolute cell density. In addition, the consistent findings in nearest-neighbor distance analyses, which are less dependent on cell density, support that the observed spatial associations are not solely driven by cellular density but rather reflect a specific immune niche.

Transcriptomic profiling also revealed that high-interaction tumors showed increased expression of IFNG and chemokine genes but reduced expression of antigen presentation-related genes such as HLA-B and RFX5. This pattern suggests that immune activation and tumor-intrinsic immune evasion may occur concurrently within the same niche, consistent with cancer immunoediting [31,32,33]. Accordingly, the high-interaction niche may represent a functional equilibrium in which activation and suppression coexist; in this context, a combined PD-L1 and VEGF blockade may have the potential to modulate this balance and reorient antitumor immunity [34,35]. The selection of a 25 µm radius is biologically plausible, as it approximates one to two cell diameters, considering that lymphocytes are typically 7–15 µm and macrophages 15–20 µm in diameter [36,37]. In addition, PD-1/PD-L1-mediated immune regulation requires close membrane proximity, supporting the use of this distance to capture functionally relevant interactions [19]. This threshold is also consistent with prior spatial immunology studies using distances of approximately 20–30 µm [24,38].

A higher PD-L1(+) TAM–CD8(+) T cell interaction variable was also associated with increased proportions of GZMB- and TIM3-expressing CD8(+) T cells. Previous studies have shown that CD8(+) T cells exposed to chronic tumor antigens can retain effector functions while expressing exhaustion-related markers, described as activated but dysfunctional or effector-like exhausted CD8(+) T cells [39,40,41,42]. These observations support the view that activation and exhaustion represent a continuous phenotypic spectrum rather than mutually exclusive states [42,43]. Atezo+Bev has been reported to modulate the tumor microenvironment through PD-L1 and VEGF inhibition, potentially enhancing immune cell interactions, whereas such immune-dependent spatial effects may be less prominent with lenvatinib [4,9,44,45,46]. Furthermore, the integration of systemic therapy with locoregional treatments, such as transarterial chemoembolization (TACE) or thermal ablation (e.g., microwave or ultrasound-guided ablation), remains a major clinical strategy. These combined approaches may induce immunogenic cell death and local environmental remodeling, potentially enhancing the recruitment and spatial clustering of CD8(+) T cells to form the favorable niches identified in this study. Looking forward, the clinical implementation of such spatial immune profiling could be significantly accelerated by the integration of artificial intelligence (AI) in digital pathology. As recently highlighted, AI-driven image analysis enables the automated and high-throughput evaluation of whole-slide images, overcoming the labor-intensive nature of manual scoring [47]. Applying AI frameworks to HCC could standardize the measurement of interaction variables, facilitating their use as routine predictive biomarkers in clinical practice. The selection of a 25 µm radius is biologically plausible, as it approximates one to two cell diameters, considering that lymphocytes are typically 7–15 µm and macrophages 15–20 µm in diameter [36,37]. In addition, PD-1/PD-L1–mediated immune regulation requires close membrane proximity, supporting the use of this distance to capture functionally relevant interactions [19]. This threshold is also consistent with prior spatial immunology studies using distances of approximately 20–30 µm [24,38].

This study has limitations. It was conducted at a single institution with a limited sample size, and analyses were based on static tissue sections at predefined treatment time points, precluding the assessment of temporal immune dynamics within the same lesion. Although radiological evaluations were performed according to institutional protocols, variations in imaging intervals inherent to retrospective studies may have influenced the timing of response assessment. The present analyses were based on pretreatment biopsy specimens and therefore represent a static snapshot of the tumor immune microenvironment, which may not fully capture the dynamic changes induced by Atezo+Bev therapy. These findings should be interpreted as reflecting the baseline immune contexture rather than fixed determinants of treatment response. Longitudinal analyses using on-treatment biopsy specimens will be essential to elucidate the dynamic remodeling of the tumor immune microenvironment during therapy. Non-invasive approaches such as liquid biopsy, including circulating tumor DNA (ctDNA) and cytokine profiling, may provide complementary insights into treatment-induced immune dynamics. In addition, the mechanisms underlying this spatial organization were not directly examined. In addition, biopsy-based spatial analysis may not fully capture the intratumoral immune heterogeneity, and sampling bias related to biopsy location cannot be completely excluded. Because a targeted gene panel was used, the analysis was limited to pre-selected genes, which may bias the pathway enrichment results. Comprehensive approaches such as whole-transcriptome sequencing, single-cell RNA sequencing, or spatial transcriptomics will be required to fully resolve cellular crosstalk and validate the proposed adaptive resistance niche. Although these analyses support the robustness of our spatial metrics, more advanced density-independent spatial statistics, such as Ripley’s K function, were not applied in this study and warrant further investigation. In addition, internal validation demonstrated that the model retained reasonable discriminative ability after correction for optimism; however, the limited number of cases and events may affect the stability of these estimates. Given the limited number of events (*n* = 15), statistical power was estimated based on the observed effect sizes. The power for vascular invasion (HR = 4.252) was approximately 0.83, indicating adequate power, whereas the power for the interaction variable (HR = 0.837) was approximately 0.52. These post hoc estimates suggest that while the findings for major clinical factors are robust, the results regarding the interaction variable should be considered exploratory. Therefore, the present findings should be considered hypothesis-generating, and external validation in larger, independent, multi-center cohorts will be required to confirm the robustness and generalizability of this spatial biomarker. Nevertheless, consistent findings across both case-based and cohort-based analyses support the robustness of our conclusions.

## 5. Conclusions

Collectively, strong spatial interactions between PD-L1(+) TAMs and CD8(+) T cells within the tumor immune microenvironment of HCC may drive the formation of a distinct immune state in which antitumor immune activation is tightly counterbalanced by immunosuppressive programs. This spatially defined tumor immune context is closely associated with responsiveness to Atezo+Bev and may represent an important molecular and immunological basis for advancing our understanding of tumor immune responses in HCC.

## Figures and Tables

**Table 1:** Characteristics of patients with hepatocellular carcinoma treated with atezolizumab plus bevacizumab and lenvatinib.

Characteristics	Atezolizumab + Bevacizumab (*n* = 23)	Lenvatinib (*n* = 20)	*p* Value
Age, median (IQR), years	75 (64–79)	73 (63–79)	0.758 *
Gender, male/female, *n*	20/3	15/5	0.440 ^†^
ECOG PS, 0/1/2/3/4, *n*	21/2/0/0/0	18/2/0/0/0	>0.999 ^†^
Etiology, HBV/HCV/NBNC, *n*	3/5/15	2/5/13	0.935 ^‡^
PLT, ×10^9^/L, median (IQR)	162 (142–202)	162 (129–258)	0.669 *
PT, INR, median (IQR)	1.04 (0.98–1.14)	1.05 (0.97–1.10)	0.668 *
ALB, g/dL, median (IQR)	3.5 (3.1–3.8)	3.7 (3.4–3.8)	0.256 *
T-bil, mg/dL, median (IQR)	0.9 (0.6–1.1)	0.8 (0.6–1.1)	0.516 *
Child–Pugh score, 5/6/7, *n*	9/6/8	12/6/2	0.935 ^‡^
Modified ALBI grade, 1/2a/2b/3, *n*	3/8/10/2	4/8/7/1	0.147 ^‡^
ALT, IU/L, median (IQR)	23 (18–35)	29 (18–37)	0.842 *
AFP, ng/mL, median (IQR)	18.5 (5.0–277.0)	20.9 (4.4–80.5)	0.871 *
DCP, mAU/mL, median (IQR)	173 (40–1244)	208 (50–655)	0.243 *
Maximum tumor size, cm, median (IQR)	4.0 (2.9–8.9)	5.0 (2.8–10.1)	0.871 *
Number of tumors, 1/2/3+, *n*	2/2/19	3/4/13	0.408 ^‡^
Vascular invasion, absent/present, *n*	17/6	12/8	0.515 ^†^
BCLC stage, A/B/C, *n*	0/12/11	0/8/12	0.544 ^‡^
Extrahepatic metastasis, *n*			
None	14	13	>0.999 ^†^
Lymph node	4	3	
Bone	3	0	
Lung	0	1	
Adrenal gland	0	2	
Lymph node, bone	1	0	
Lymph node, lung	1	0	
Lymph node, lung, adrenal gland	0	1	
Prior systemic therapy, *n*			
None	15	9	0.228 ^†^
Sorafenib	0	0	
Lenvatinib	5	―	
Atezolizumab plus bevacizumab	―	7	
HAIC	1	3	
Sorafenib, HAIC	1	1	
Lenvatinib, HAIC	1	0	
Observation period, median, days	435	436	0.503 *

AFP, a-fetoprotein; BCLC stage, Barcelona Clinic Liver Cancer stage; DCP, des-gamma-carboxy prothrombin; HAIC, hepatic arterial infusion chemotherapy; IQR, interquartile range; NBNC, nonB-nonC. *, Mann–Whitney U test. ^†^, Fisher’s exact test. ^‡^, Chi-square test.

**Table 2:** Characteristics of patients with HCC who underwent hepatectomy.

Variable	Value
Age, median (IQR), years	65 (61–71)
Sex, male/female, *n*	7/1
Etiology, HBV/HCV/NBNC, *n*	1/3/4
PLT, ×10^9^/L, median (IQR)	169 (121–193)
PT, %, median (IQR)	88.4 (80.2–104.1)
ALT, IU/L, median (IQR)	29 (17–43)
ALB, g/dL, median (IQR)	4.0 (3.7–4.1)
T-bil, mg/dL, median (IQR)	1.0 (0.8–1.4)
Child–Pugh score, 5/6/7, *n*	7/0/1
Modified ALBI grade, 1/2a/2b/3, *n*	4/2/2/0
AFP, ng/mL, median (IQR)	5.7 (4.2–8.1)
DCP, mAU/mL, median (IQR)	42 (31–210)
Maximum tumor size, mm, median (IQR)	26 (22–41)
Tumor multiplicity, single/multiple, *n*	8/0
Tumor differentiation, well/moderate/poor, *n*	3/4/1
Vascular invasion	
Vp, 0–1/2–3, *n*	8/0
Vv, 0–1/2, *n*	8/0
Va, 0–1/2, *n*	8/0
BCLC stage, 0/A/B/C, *n*	0/8/0/0
Fibrosis, 0/1/2/3/4, *n*	0/2/2/1/3

AFP, a-fetoprotein; ALB, albumin; ALBI, albumin-bilirubin; ALT, alanine aminotransferase; BCLC stage, Barcelona Clinic Liver Cancer stage; DCP, des-gamma-carboxy prothrombin; HBV, hepatitis B virus; HCV, hepatitis C virus; IQR, interquartile range; NBNC, nonB-nonC; PLT, platelet; PT, prothrombin time; T-bil, total bilirubin.

**Table 3:** Univariate and multivariate analysis of factors associated with progression-free survival of patients with hepatocellular carcinoma treated with atezolizumab plus bevacizumab.

		Univariate Analysis		Multivariate Analysis	
Variables	Patients (*n* = 23)	*p* Value	Hazard Ratio	95% CI	*p* Value
Tumor biopsy					
Interaction variable; PD-L1(+) TAM and CD8(+) T cells, (<median/≥median)	11/12	0.001	0.8373	0.6870–0.9773	0.022
Counts of CD8(+) T cells, (<median/≥median)	12/11	0.376			
Counts of PD-L1(+) TAM, (<median/≥median)	12/11	0.956			
Tumor differentiation (well/moderate, poor)	11/12	0.273			
Clinical features					
Age, y, (≤75/>75)	11/12	0.619			
Gender (male/female)	20/3	0.244			
Etiology (HBV, HCV/NBNC)	8/15	0.231			
Child–Pugh score, (5/6/7)	9/14	0.557			
mALBI grade, (1/2a/2b/3)	11/12	0.610			
AFP, ng/mL, (<13.4/≥13.4)	10/13	0.219			
DCP, mAU/mL, (<100/≥100)	9/14	0.017	1.000	0.9999–1.000	0.307
Maximum tumor size, cm, (<4/≥4)	10/13	0.327			
Tumor number, (<3/≥3)	4/19	0.445			
Vascular invasion (absent/present)	6/17	0.030	4.252	1.060–17.44	0.042
Extrahepatic metastasis (absent/present)	17/6	0.497			
BCLC stage (B/C)	12/11	0.332			
Prior systemic therapy (None or HAIC/TKI)	17/6	0.119			
Serum cytokines					
IL-8, (<median/≥median)	12/11	0.637			
IL-1β, (<median/≥median)	13/10	0.486			
IL-6, (<median/≥median)	12/11	0.607			
IL-10, (<median/≥median)	12/11	0.214			
TNF, (<median/≥median)	12/11	0.723			
IL-12p70, (<median/≥median)	12/11	0.156			

AFP, a-fetoprotein; BCLC stage, Barcelona Clinic Liver Cancer stage; CTC, circulating tumor cell; DCP, des-gamma-carboxy prothrombin; HAIC, hepatic arterial infusion chemotherapy; H-score, histological score; IHC, immunohistochemistry; mALBI, modified albumin-bilirubin; NBNC, nonB-nonC; PD-L1, programmed death ligand 1; qRT-PCR, quantitative reverse-transcription polymerase chain reaction; TKI, tyrosine kinase inhibitor.

## Data Availability

The data of the current study are available from the corresponding author upon reasonable request. The data are not publicly available due to privacy and ethical reasons.

## Supplementary Materials

The following supporting information can be downloaded at https://www.mdpi.com/article/10.3390/cancers18091422/s1. Supplementary Figure S1: Quantification of spatial interactions between PD-L1(+) TAMs and CD8(+) T cells. Supplementary Figure S2: Robustness of interaction variables across multiple distance thresholds. A correlation matrix comparing interaction variables measured at four spatial thresholds (15, 25, 30, and 50 µm). Diagonal panels show histograms of each variable with overlaid kernel density estimates (red lines). Lower triangular panels display bivariate scatter plots with fitted linear regression lines (red). Upper triangular panels present Spearman’s rank correlation coefficients (ρ), with font size proportional to the correlation strength. *** *p* < 0.001. Supplementary Figure S3: Forest plot of the interaction variable at different spatial thresholds. Cox proportional hazards regression analysis was performed using four spatial distance thresholds (15, 25, 30, and 50 µm). Hazard ratios (black squares) and 95% confidence intervals (horizontal error bars) for progression-free survival (PFS) are shown for each distance. The vertical line indicates the null value (HR = 1.0). Supplementary Table S1. Lists of reagents and resources. Supplementary Table S2. Univariate analysis of factors associated with the progression-free survival of patients with hepatocellular carcinoma treated with lenvatinib.
