# Supplementary material for: Spatial Proximity Between PD-L1(+) Tumor-Associated Macrophages and CD8(+) T Cells Influences Response to Atezolizumab Plus Bevacizumab in Hepatocellular Carcinoma
Source: Cancers (Basel). 2026 Apr 29;18(9):1422. doi: 10.3390/cancers18091422 (PMC13162669; doi:10.3390/cancers18091422)
Supplement: Supplementary file 1 [file cancers-18-01422-s001.zip › Supplementary Tables.pdf]

Supplementary Table S1. Lists of reagents and resources

REAGENT or RESOURCE	SOURCE	IDENTIFIER
Antibodies		
anti-human CD68 (clone KP1)	Gene Tex	Cat# GTX73643; RRID: AB_375045
anti-human, mouse CD163 (clone EPR19518)	abcam	Cat# ab182422; RRID: AB_2753196
anti-human CD8 (clone 1G2B10)	Proteintech	Cat# 66868-1-Ig; RRID: AB_2882205
anti-human, mouse PD-L1 [SP142] (clone SP142)	abcam	Cat# ab228462; RRID: AB_2827816
anti-human, mouse TIM3 (clone EPR22241)	abcam	Cat# ab241332; RRID: AB_2888936
anti-human, mouse Granzyme B (clone EPR22645-206)	abcam	Cat# ab255598; RRID: AB_2860567
Goat Anti-Rat IgG H&L (HRP) preadsorbed	abcam	Cat# ab7097; RRID: AB_955411
Goat Anti-Rabbit IgG H&L (HRP) preadsorbed	abcam	Cat# ab7090; RRID: AB_955417
Chemicals, peptides, and recombinant proteins		
ProLong™ Diamond Antifade Mountant	Thermo Fisher Scientific	P36961
Bond TM Epitope Retrieval 1	Leica Biosystems	AR9961
Bond TM Epitope Retrieval 2	Leica Biosystems	AR9640
Histofine SimpleStain MAX PO (MULTI)	Nichirei	424151
Histofine SimpleStain mouse MAX PO (M)	Nichirei	414131
Histofine SimpleStain mouse MAX PO (R)	Nichirei	414341

Critical commercial assays		
Opal 6-Plex Manual Detection Kit	Akoya Biosciences	NEL811001KT
BD™ Cytometric Bead Array (CBA) Human Inflammatory Cytokine Kit I	BD Biosciences	551811
Software and algorithms		
R: The Project for Statistical Computing	N/A	https://www.r-project.org/
RStudio	N/A	https://posit.co/download/rstudio-desktop/
inForm	Akoya Biosciences	https://www.akoyabio.com/phenoimager/inform-tissue-finder/
phenoptrReports & phenoptr	Akoya Biosciences	https://www.akoyabio.com/phenoimager/phenoptrreports-phenoptr/
Prism	GraphPad Software	https://www.graphpad.com/

Supplementary Table S2. Univariate analysis of factors associated with progression-free survival of patients with hepatocellular carcinoma treated with Lenvatinib

Variables	Patients (n = 20)	Univariate analysis p value
**Tumor biopsy**		
Interaction valiable; PD-L1(+) TAM and CD8(+) T cells, (< median/ ≥ median)	10/10	0.298
Counts of CD8(+) T cells, (< median/ ≥ median)	10/10	0.341
Counts of PD-L1(+) TAM, (< median/ ≥ median)	9/11	0.495
Tumor differentiation, (well/moderate, poor)	9/11	0.104
**Clinical features**		
Age, y, (≤ 75/ > 75)	10/10	0.184
Gender, (male/female)	15/5	0.919
Etiology, (HBV, HCV/NBNC)	7/13	0.536
Child-Pugh score, (5/6,7)	12/8	0.751
mALBI grade, (1,2a/2b,3)	12/8	0.610
AFP, ng/mL, (< 13.4/ ≥ 13.4)	9/11	0.081
DCP, mAU/mL, (< 100/ ≥ 100)	7/13	0.563
Maximum tumor size, cm, (< 4/ ≥ 4)	8/12	0.108
Tumor number, (< 3/ ≥ 3)	7/13	0.986
Vascular invasion, (absent/present)	8/12	0.101
Extrahepatic metastasis, (absent/present)	11/9	0.497
BCLC stage (B/C)	8/12	0.050
Prior systemic therapy, (None or HAIC/ ICI-based systemic therapy)	9/11	0.119
**Serum cytokines**		
IL-8, (< median/ ≥ median)	10/10	0.079
IL-1β, (< median/ ≥ median)	10/10	0.460
IL-6, (< median/ ≥ median)	10/10	0.461
IL-10, (< median/ ≥ median)	10/10	0.122
TNF, (< median/ ≥ median)	10/10	0.871
IL-12p70, (< median/ ≥ median)	10/10	0.086

Abbreviations: AFP, a-fetoprotein; BCLC stage, Barcelona Clinic Liver Cancer stage;

CTC, circulating tumor cell; DCP, des-gamma-carboxy prothrombin; HAIC, hepatic arterial infusion chemotherapy; H-score, histological score; ICI, immune checkpoint inhibitor; IHC, immunohistochemistry; mALBI, modified albumin-bilirubin; NBNC, nonB-nonC; PD-L1, programmed death ligand 1; qRT-PCR, quantitative reverse-transcription polymerase chain reaction.
