# Supplementary figures and images for: Spatial Proximity Between PD-L1(+) Tumor-Associated Macrophages and CD8(+) T Cells Influences Response to Atezolizumab Plus Bevacizumab in Hepatocellular Carcinoma
Source: Cancers (Basel). 2026 Apr 29;18(9):1422. doi: 10.3390/cancers18091422 (PMC13162669; doi:10.3390/cancers18091422)

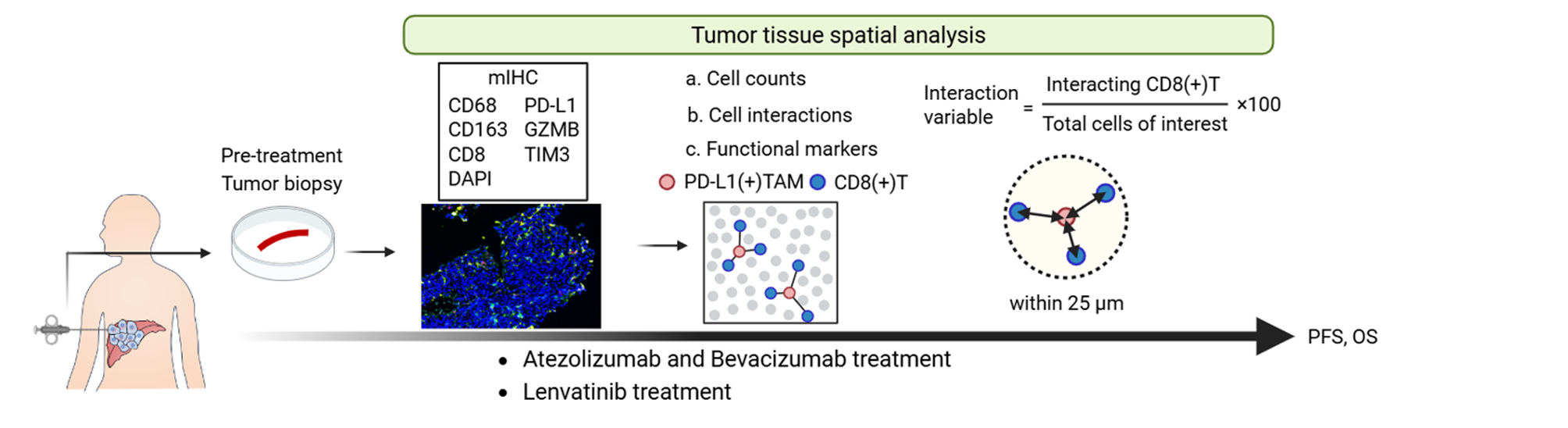

Supplement: Supplementary file 1 [file cancers-18-01422-s001.zip › Supplementary Figure S1.tif]

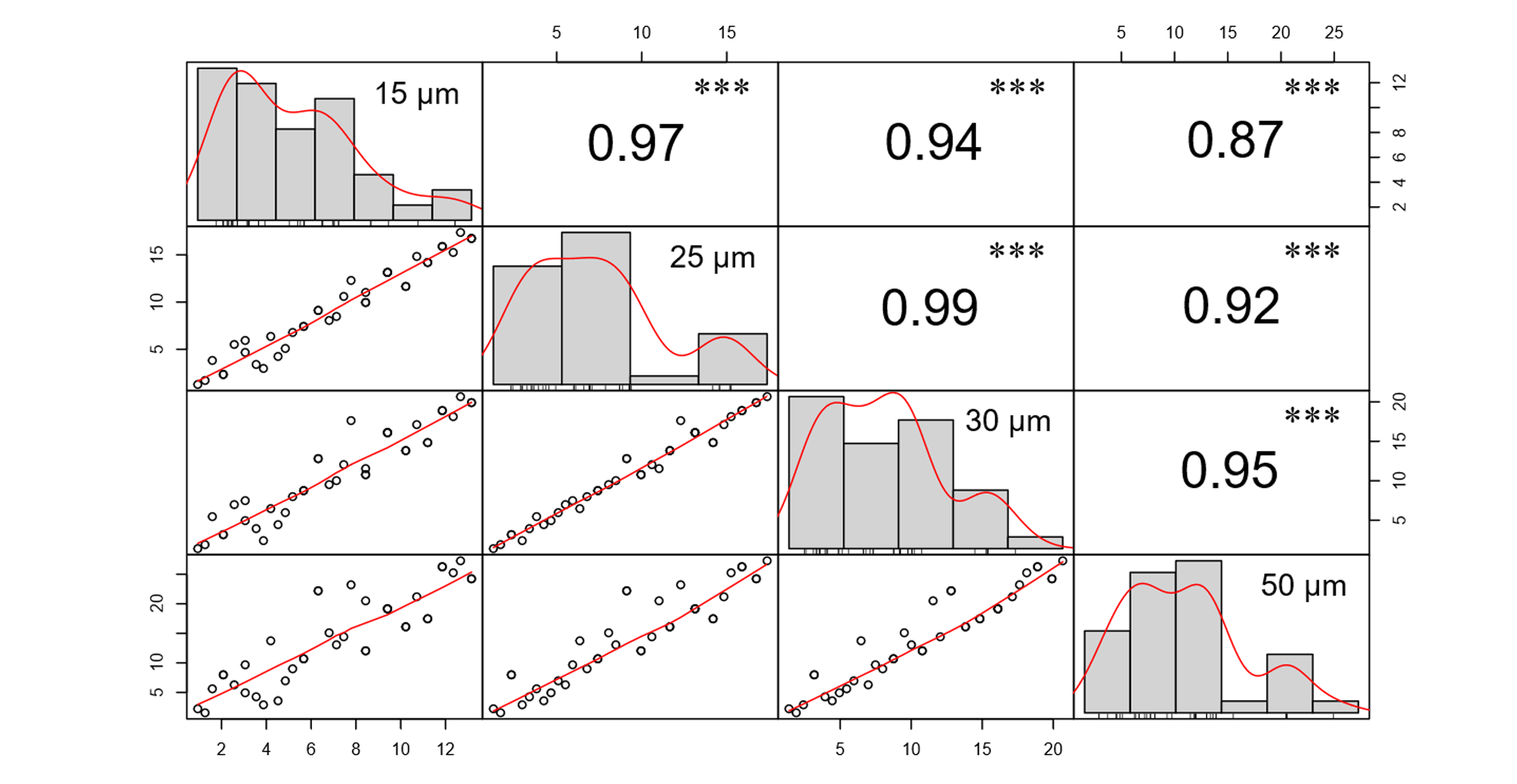

Supplement: Supplementary file 1 [file cancers-18-01422-s001.zip › Supplementary Figure S2.tif]

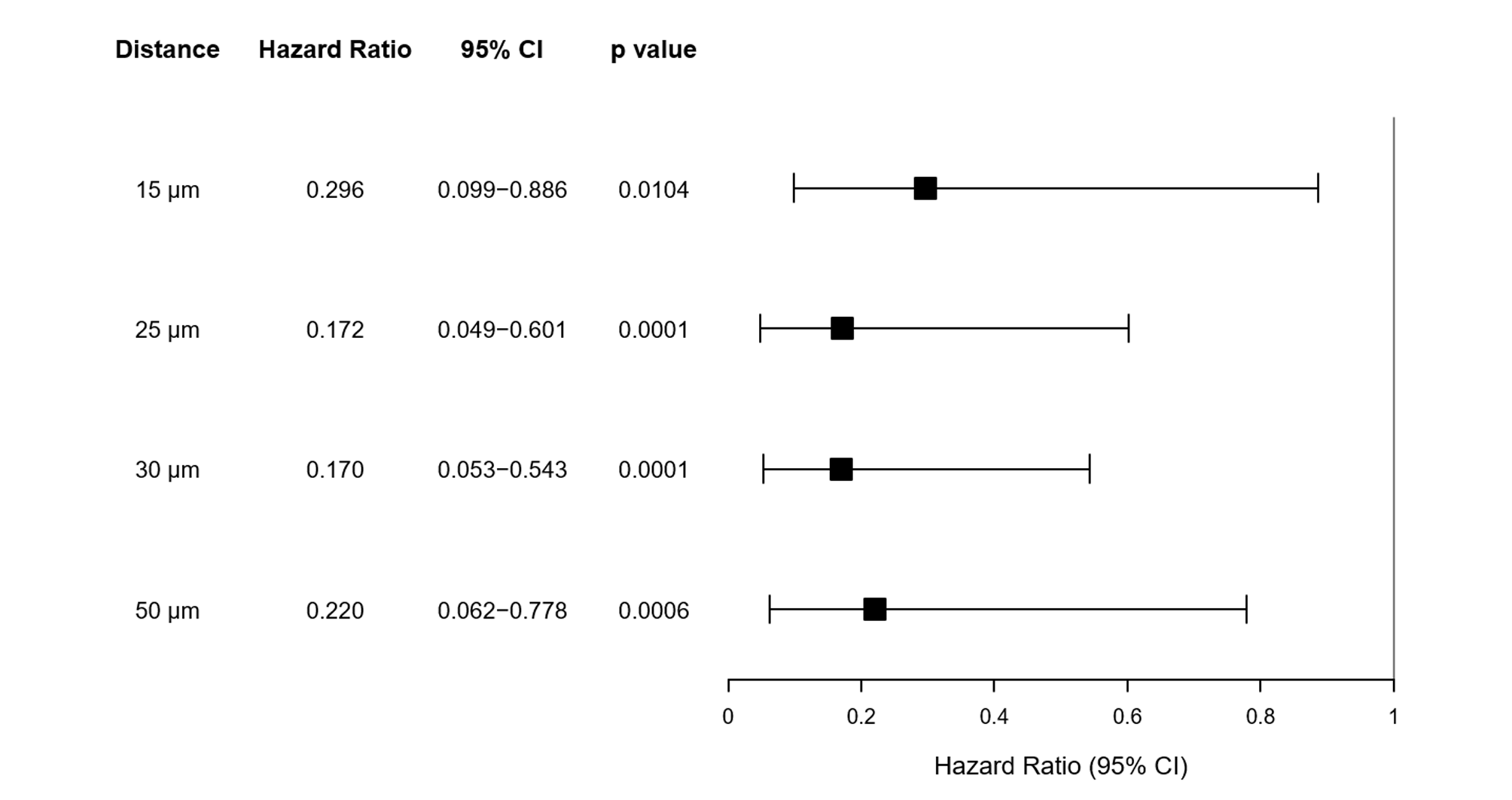

Supplement: Supplementary file 1 [file cancers-18-01422-s001.zip › Supplementary Figure S3.tif]
